# Feasibility of creating estimates of left ventricular flow-volume dynamics using echocardiography

**DOI:** 10.1186/1476-7120-4-40

**Published:** 2006-10-31

**Authors:** Emil Söderqvist, Peter Cain, Britta Lind, Reidar Winter, Jacek Nowak, Lars-Åke Brodin

**Affiliations:** 1 Division of Medical Engineering, Department of Laboratory Medicine, Karolinska Institutet, Karolinska University Hospital, Stockholm, Sweden; 2 Division of Clinical Physiology, Department of Laboratory Medicine, Karolinska Institutet, Karolinska University Hospital, Stockholm, Sweden

## Abstract

**Background:**

This study explores the feasibility of non-invasive assessment of left ventricular volume and flow relationship throughout cardiac cycle employing echocardiographic methods.

**Methods:**

Nine healthy individuals and 3 patients with severe left-sided valvular abnormalities were subject to resting echocardiography with automated endocardial border detection allowing real-time estimation of left ventricular volume throughout the cardiac cycle. Global and regional (6 different left ventricular segments) estimates of flow-volume loops were subsequently constructed by plotting acquired instantaneous left ventricular 2-D area data (left ventricular volume) vs. their first derivatives (flow).

**Results:**

Flow-volume loop estimates were obtainable in 75% of all echocardiographic images and displayed in normal individuals some regional morphological variation with more pronounced isovolumic events in the paraseptal segments and significantly delayed maximal systolic flow paraapically. In patients with aortic stenosis, maximal systolic flow occurred at a lower estimated left ventricular systolic volume whereas in mitral stenosis, maximal diastolic flow was observed at a higher estimated left ventricular diastolic volume. Aortic regurgitation caused a complex alteration of the estimated flow-volume loop shape during diastole.

**Conclusion:**

Non-invasive assessment of left ventricular flow-volume relationship with echocardiography is technically feasible and reveals the existence of regional variation in flow-volume loop morphology. Valvular abnormalities cause a clear and specific alteration of the estimates of the normal systolic or diastolic flow-volume pattern, likely reflecting the underlying pathophysiology.

## Background

Flow-volume measurement is well established as a clinical measure of lung function through its ability to demonstrate the dynamic nature of an underlying obstructive or restrictive process [1]. Although there are differences in the underlying dynamics, potentially, the same measure may be applied to the function of the left ventricle as the left ventricular volume-blood flow relationship throughout the cardiac cycle may be changed during altered loading conditions or in certain diseased states of the cardiac valves, myocardium or pericardium which in turn may also be restrictive or obstructive in nature. Normal systolic flow-volume relationships may, for example, be disturbed by aortic or sub-aortic obstruction while diastolic flow-volume relationships may be affected by mitral stenosis, myocardial restrictive processes or significant left ventricular hypertrophy.

Non-invasive estimation of left ventricular flow-volume loops could be obtained using transthoracic echocardiography with continuous information of the left ventricular volume changes throughout the cardiac cycle. Since the left ventricular 2-dimensional (2-D) area as used in the Simpson's rule algorithm for the calculation of left ventricular ejection fraction [2-4] is related to the left ventricular blood volume, the first derivative of such a volume index during the cardiac cycle would relate to the rate of change of the ventricular volume entering and exiting the left ventricle [5]. Hence, the first derivative of the 2-D left ventricular area would constitute an estimate of left ventricular blood flow. The first derivative could then be graphically presented together with corresponding 2-D area representing left ventricular volume throughout the cardiac cycle to create an estimate of left ventricular flow-volume loop. Furthermore, the left ventricular area could be divided into several sections to reflect segmental function of the left ventricle with the first derivative of a given region reflecting segmental volume displacement.

The aim of this study was to develop and explore the feasibility of a non-invasive estimation of left ventricular flow-volume relationship throughout the cardiac cycle with echocardiography according to the conceptual framework outlined above. The secondary aim was to identify normal ranges of global systolic and diastolic flow estimates and to assess the normal contributions of different left ventricular segments. In addition, examples of estimated flow-volume loops are provided in patients with left ventricular valvular disease to illustrate the applicability of such a non-invasive approach in these conditions.

## Methods

### Design

The study population consisted of nine individuals (5 men and 4 women), 39 ± 23 years old, without any signs of coronary artery disease and normal left ventricular function, and 3 patients (2 men, 84 and 50 years old, and 1 woman, 75 years old) with severe valvular abnormalities (aorta stenosis, aorta insufficiency, and mitral stenosis, respectively) for demonstration of potential pathological flow-volume loop morphology. All the study participants were subject to resting echocardiography. Individuals with poor image quality, complex atrial or ventricular arrhythmias, or previous revascularisation were excluded from the study. The study was approved by the ethics committee at Karolinska Institutet – Huddinge University Hospital and all the study subjects gave their informed consent to participate.

### Image acquisition

Echocardiographic imaging was performed with the subjects in left lateral position using a commercially available system (Aloka PhD Prosound SSD 5500, Aloka, Tokyo, Japan). Images were obtained in the three standard apical views (four chamber, long axis, two chamber) using a standard 3 MHz transducer. Two-dimensional gray scale images with real-time left ventricular endocardial detection were stored digitally for subsequent off-line analysis.

### Real time detection of left ventricular volume

**Figure 1:**
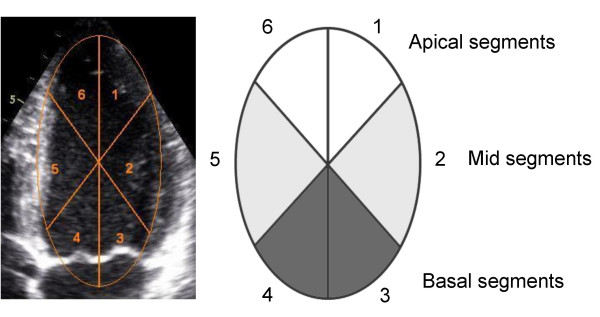
Left ventricular 4-chamber view (left) with the ellipsoid region of interest applied. The region of interest is divided into six segments. For each view the segments were grouped into apical, mid, and basal segments (right).

Using commercially available software (ASMA, Aloka, Tokyo, Japan), an ellipsoid region of interest (ROI) was defined so that the left ventricular endocardium could be encapsulated for the whole cardiac cycle while taking care to avoid inclusion of data external to the left ventricular cavity. The left ventricular endocardium was then automatically detected in real time for each frame throughout the cardiac cycle (100 frames/s). The left ventricular cavity thus delineated was subsequently displayed 'filled in' with orange pixels (Figure 1). Using the quantitative features of the ASMA software the cardiac cycle volume characteristics based on this area of orange pixels was then obtained in graphical form for the whole of the left ventricle as well as for six regional segments of the ROI (Figure 1). As can be seen from Figure 1, these segments of ROI varied from the accepted standard of segmentation of the left ventricle [6] and, in particular, the regions of interest of the basal segments crossed the mitral valve plane in the apical views. The six segments did however allow separations of data into basal, mid-level, and apical regions. All images were stored as digital cineloops in DICOM format for reference of segment location while global and regional data characteristics were also exported in a hexadecimal numerical format to a standard PC for off-line data analysis.

### Development of echocardiographically derived estimates of left ventricular flow-volume loops and data analysis

**Figure 2:**
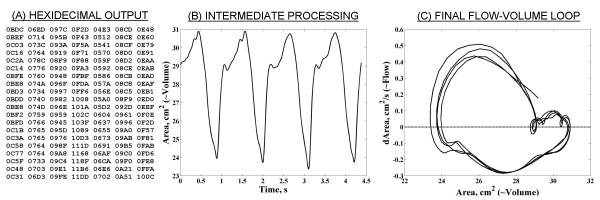
Derivation of the flow-volume estimates loop. (A) Seven columns of hexadecimal volume information (six segments, one global) are exported for each frame of the cardiac cycle. These values are standardised by a factor supplied by the automated software. (B) All values converted to decimal format are plotted as area (estimate of left ventricular volume) versus time. (C) The area ("volume") is the plotted against its first derivative (indicative of ventricular flow) to form the final flow-volume estimates loop.

Hexadecimal numerical data (Figure 2A) were converted to decimal format and imported to Matlab (Version 6.5, TheMathWorks Inc., Natick, MA, U.S.A.). The values were then adjusted by a calibrating factor exported from the ASMA acquisition software and expressed in cm^2^. After low grade temporal filtering using polynomial fitting [7], graphs of both global and segmental area versus time were established in each cardiac view (Figure 2B). The area (estimate of left ventricular volume) was then plotted against its first derivative (area change – indicative of ventricular flow) to create loop plots for the whole ventricle and for each segment, called global and segmental (regional) estimates of flow-volume loops, respectively (Figure 2C). The loops created in this way lack time scale and, instead, have the estimate of left ventricular volume (left ventricular 2-D area) as x-axis. Consequently, expressions pointing to early/late diastolic/systolic maximum flows mean in reality maximum flows at small/large left ventricular volumes, respectively.

**Figure 3:**
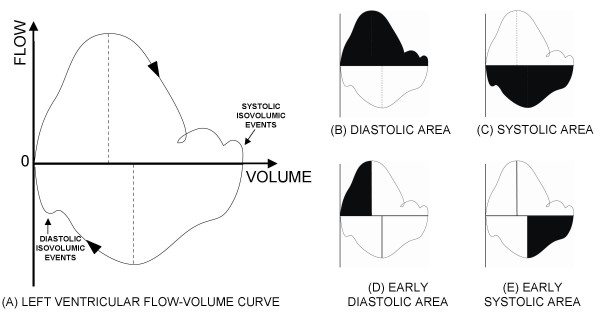
(A) A schematic diagram of a left ventricular global flow-volume estimates loop. The flow-volume estimates loop proceeds in a clock wise direction. Minimum and maximum volumes are readily identified as well as maximum systolic and maximum diastolic flow. Isovolumic systolic and diastolic events are also identifiable. Integrals of the flow-volume estimates loop are also shown representing the (B) diastolic, (C) systolic, (D) early diastolic, and (E) early systolic volume changes.

Following variables were measured in the echocardiographically derived global and segmental estimates of flow-volume loops as schematically presented in Figure 3:

**V**_**e**max_**: **Estimate of maximal left ventricular volume during the cardiac cycle (i.e. left ventricular area at end-diastole; Figure 3A).

**V**_**e**min_**: **Estimate of minimal left ventricular volume during the cycle (i.e. left ventricular area at end-systole; Figure 3A).

**F**_**e**max_diast**: **Estimate of maximal diastolic left ventricular flow during the cardiac cycle (Figure 3A).

**F**_**e**max_syst**: **Estimate of maximal systolic left ventricular flow during the cardiac cycle (Figure 3A).

**A**diast**: **Area under flow-volume curve (NB, not left ventricular image area) during diastole (Figure 3B).

**A**syst**: **Area under flow-volume curve (NB, not left ventricular image area) during systole (Figure 3C).

**A**ediast**: **Area under flow-volume curve during early diastole (prior to **F**_**e**max_diast; Figure 3D).

**A**esyst**: **Area under flow-volume curve during early systole (prior to **F**_**e**max_syst; Figure 3E).

**TA**ediast**:**Time interval (expressed as a fraction of diastole) for **A**ediast, i.e. timing of **F**_**e**max_diast.

**TA**esyst**: **Time interval (expressed as a fraction of diastole) for **A**esyst, i.e. timing of **F**_**e**max_syst.

Although the isovolumic systolic and diastolic events in flow-volume loops could be delineated (as labeled in Figure 3A), the approach to their quantification is unclear at this time and, consequently, these events were not quantified.

### Statistical analysis

All statistical analyses was performed using standard statistical software (SPSS version 11.01). Continuous data are presented as mean and standard deviation while categorical data are presented as frequency. Mean values for each variable were compared by independent t-test and ANOVA. Frequency analysis between categories was achieved with the χ^2 ^test.

## Results

### Echocardiographic data and feasibility

With the off-line software used, the time required to import, convert and analyse data, and to produce graphical presentation was 3–5 minutes.

All individuals in the normal group had normal resting ventricular wall motion. Of the 36 echocardiographic images available for acquisition in this group, 28 (75%) provided sufficient resolution to allow adequate automated endocardial border detection. The 4-chamber view was most favorable in this respect with all the images obtained in this view allowing adequate delineation of the left ventricular cavity whereas the percentage of qualitatively satisfactory images in the two other views was lesser (67% of the images in apical 2-chamber view and 55% of the images in apical long axis view, p = 0.001). There were several reasons for exclusion of images from analysis, sub-optimal image quality being the most prominent factor. Another important reason was inability to obtain the region of interest closely enough to the shape of the left ventricular endocardium throughout the cardiac cycle which fact would result in underestimation of global and regional data and their contamination with extraneous signal components from outside the left ventricular cavity. An irregular RR-interval of the cardiac cycle and poor breath holding resulted in poor reproducibility of the flow-volume loops and the corresponding images were therefore excluded from the analysis as well.

### Estimated global and regional flow-volume curves

**Figure 4:**
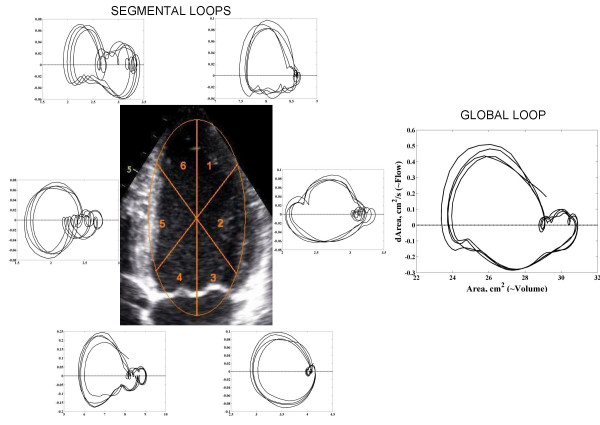
Global and segmental left ventricular flow-volume estimates loops derived from image obtained in 4-chamber view. Note the variation in the forms of segmental flow-volume estimates loops depending on segment location.

Figure 3 provides a schematic diagram of an estimated global left ventricular flow-volume loop whereas typical estimated regional curves and a typical global flow-volume loop from a healthy individual are shown in Figure 4.

As illustrated in Figure 3A, the loop proceeds in clockwise direction with positive values for flow into the left ventricle during diastole and negative flow values for flow out of the ventricle during the systolic ejection. As could be expected, the loop indicates maximum left ventricular volume at end-diastole and minimum volume at end-systole. Besides the main systolic and diastolic cardiac events (systolic ejection, diastolic E- and A-waves) that are clearly identifiable within the estimated flow-volume loop, the systolic and diastolic isovolumic events can be identified as well.

Estimated regional flow-volume loops were more complex in their forms in comparison to the global ones and there occurred differences in curve morphology between the loops from different left ventricular cavity segments (Figure 4). As can be seen from Figure 4, the regional loops tend to display, in general, somewhat more pronounced isovolumic changes in the septal segments (segments 4–6) then in the free wall segments (segments 1–3). In turn, estimated volume changes during rapid diastolic filling and systolic ejection appear to be more pronounced in the free wall segments.

### Normal ranges of estimated global flow-volume loop variables

Table 1 shows the mean values for all variables measured in global echocardiographically derived estimates of flow-volume loops in each echocardiographic view. As can be seen from the table, the values of estimated maximal and minimal left ventricular volume as well as the values of maximal systolic and diastolic flow and their timings were similar in all apical views. The absolute values of estimates of peak diastolic and systolic flow were also similar, and this was consistent across all views. The integral of the estimated flow-volume loop during the diastolic and systolic phases (**A**diast and **A**syst, respectively) allowed the assessment of the dynamics of blood movement into and out of the ventricle, respectively. In currently studied healthy individuals without any significant intra-ventricular shunt, this integral was roughly the same for both the diastolic and systolic components of the estimated flow volume loop. No differences in this respect occurred between the different apical views either.

**Table 1:** Variables extracted from echocardiographically derived global flow-volume curves. Mean values ± SD are presented.

**Variable**		**Echocardiographic view**				
		
		**Apical 4-chamber**	**Apical 2-chamber**	**Apical Long axis**	**All apical**	***p***
**V**_**e**min_	[cm^2^]	17.52 ± 5.15	12.48 ± 4.53	16.44 ± 7.96	15.94 ± 6.05	0.34
**V**_**e**max_	[cm^2^]	26.20 ± 6.50	22.19 ± 7.99	24.19 ± 7.63	24.60 ± 7.03	0.61
**F**_**e**max_diast	[cm^2^/s]	0.45 ± 0.15	0.53 ± 0.15	0.37 ± 0.19	0.44 ± 0.16	0.30
**F**_**e**max_syst	[cm^2^/s]	-0.39 ± 0.12	-0.45 ± 0.19	-0.38 ± 0.15	-0.40 ± 0.14	0.71
**A**diast	[cm^4^/s]	2.69 ± 1.50	3.45 ± 2.14	2.04 ± 1.52	2.69 ± 1.68	0.40
**A**syst	[cm^4^/s]	-2.61 ± 1.48	-3.74 ± 2.91	-2.21 ± 1.39	-2.77 ± 1.89	0.41
**A**ediast	[cm^4^/s]	0.46 ± 0.15	0.39 ± 0.14	0.53 ± 0.18	0.46 ± 0.15	0.28
**A**esyst	[cm^4^/s]	-0.45 ± 0.15	-0.45 ± 0.18	-0.52 ± 0.12	-0.47 ± 0.14	0.69
**TA**ediast	-	0.30 ± 0.26	0.23 ± 0.09	0.32 ± 0.29	0.29 ± 0.23	0.80
**TA**esyst	-	0.47 ± 0.17	0.42 ± 0.18	0.42 ± 0.11	0.44 ± 0.16	0.82

### Normal ranges of estimated segmental flow-volume loop variables

With 24 regional segments available for analysis, it is not practical to show the results for all of the measured loop variables for each of these segments. Therefore, the segments were grouped before the analysis was performed. Table 2 demonstrates the regional variation for all estimated segmental flow-volume loop variables according to their regional position as a basal, mid-ventricular, or apical region within the left ventricle. The estimated segmental **V**_**e**min _values registered at the mid-ventricular level were significantly lower (p < 0.05) than those registered in the basal and apical levels. There was also a tendency toward lower **V**_**e**max _at this level but the difference did not reach the level of statistical significance. On the other hand, the estimated maximal systolic and diastolic flow values, and the integrals of the systolic and diastolic components of the estimated regional loop were not significantly different in any of the three ventricular locations considered. Interestingly, there was a delay in estimated peak systolic flow noted in the apical segments compared to the basal segments (p = 0.05). In concordance with this delay, there was also an increased early systolic integral (**A**esyst) in the regional apical loop prior to **F**_**e**max_syst (p < 0.00).

**Table 2:** Variables extracted from echocardiographically derived regional flow-volume curves. Mean values ± SD are presented.

**Variable**		Left ventricular region*				
		
		**Apical Segment 1 & 6**	**Midventricular Segment 2 & 5**	**Basal Segment 3 & 4**	**Total**	***p***
**V**_**e**min_	[cm^2^]	2.96 ± 2.16	1.71 ± 1.13	2.64 ± 2.16	2.44 ± 1.93	0.03
**V**_**e**max_	[cm^2^]	4.78 ± 2.11	3.63 ± 1.56	4.59 ± 2.84	4.32 ± 2.20	0.09
**F**_**e**max_diast	[cm^2^/s]	0.11 ± 0.06	0.11 ± 0.06	0.12 ± 0.07	0.11 ± 0.06	0.69
**F**_**e**max_syst	[cm^2^/s]	-0.09 ± 0.06	-0.11 ± 0.05	-0.10 ± 0.05	-0.10 ± 0.05	0.70
**A**diast	[cm^4^/s]	0.17 ± 0.21	0.18 ± 0.18	0.19 ± 0.16	0.17 ± 0.19	0.92
**A**syst	[cm^4^/s]	-0.16 ± 0.19	-0.18 ± 0.15	-0.16 ± 0.15	-0.17 ± 0.16	0.90
**A**ediast	[cm^4^/s]	0.44 ± 0.12	0.46 ± 0.16	0.44 ± 0.17	0.45 ± 0.15	0.84
**A**esyst	[cm^4^/s]	-0.48 ± 0.15	-0.44 ± 0.14	-0.34 ± 0.11	-0.43 ± 0.15	0.00
**TA**ediast	-	0.25 ± 0.17	0.32 ± 0.28	0.27 ± 0.26	0.28 ± 0.24	0.55
**TA**esyst	-	0.60 ± 0.18	0.52 ± 0.33	0.45 ± 0.21	0.53 ± 0.24	0.05

* as depicted in Figure 1

Despite the visual appearance of differing curve form, there were no significant differences for all estimated flow-volume loop variables between the left ventricular free wall and septal segments.

### Estimated flow volume loop form in patients with severe valvular abnormalities

**Figure 5:**
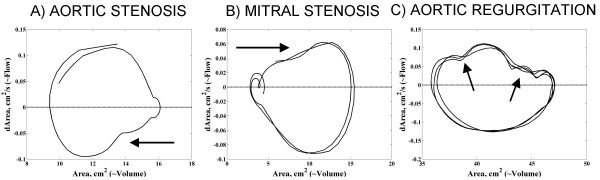
Global flow-volume estimates loops in patients with severe left sided valvular disease. (A) Note peak systolic flow occurring at a lower relative ventricular volume in systole (arrow); (B) Note peak diastolic flow at a higher relative ventricular volume in diastole (arrow); (C) Note complex diastolic flow-volume estimates relationships in diastole in a patient with aortic regurgitation with altered early and late diastolic filling (arrows).

Figure 5 shows the estimated global flow-volume loops from three patients with severe left sided cardiac valve pathologies. As can be seen from the Figure, the loop form was altered in all three cases and differed from a typical global flow-volume loop in normal individuals (cf. Figure 4).

In the presence of aortic stenosis, there was an alteration in the estimated flow-volume relationship during systole. **F**_**e**max_syst occurred at a lower estimated left ventricular volume during systole consistent with impaired left ventricular emptying.

In the case of mitral stenosis, the rate of filling of the left ventricle was notably altered and **F**_**e**max_diast appeared at a higher estimated left ventricular volume during diastole compared with the normal individuals.

Finally, in the patient with severe aortic regurgitation, a complicated estimated flow-volume relationship was seen in diastole. This complex flow-volume pattern differed from what could be seen in the estimated normal global flow-volume loop, and thus reflected altered diastolic filling of the left ventricle in this valvular abnormality.

## Discussion

This study explores the feasibility of a non-invasive assessment of left ventricular flow-volume relationship throughout the cardiac cycle using transthoracic echocardiography technique. The obtained results demonstrate that such an approach indeed can provide estimates of left ventricular flow-volume characteristics. Furthermore, software analysis of data could easily be automated and integrated in the echocardiographic equipment, thus enabling on-line presentation of the results. The estimated flow-volume loops derived from the acquired data provide physiologically intuitive information about the extent of peak diastolic and systolic blood flow, end diastolic and end systolic left ventricular volumes and their relationship to each other both in normal individuals and in patients with significant left-sided valvular pathology. Other ventricular events as, for example, the contribution of the atrial volume to diastolic filling may also be appreciated. In addition, the analysis of regional displacement of the cardiac walls suggests that there may be an asymmetrical pattern of myocardial deformation throughout the cardiac cycle that may, in part, mirror the current physiological concept of the left ventricular motion dynamics.

The reliability of presented methodology is based on certain assumptions. Firstly, the assessment of changes of global left ventricular volume in the present study was obtained by measuring left ventricular 2-D areas in echocardiographic images and the measured 2-D areas were the same as those used for calculation of left ventricular volumes and ejection fraction with Simpson's rule algorithm [2-4]. The Simpson's algorithm has been shown to perform sufficiently robustly in the clinical setting [8] and the left ventricular area measurements have been shown to reflect accurately changes in the left ventricular volume measured with conductance volumetry [5]. Hence, the currently measured left ventricular area would constitute a reasonably good estimate of left ventricular volume dynamics. However, it should be kept in mind, that the Simpson's algorithm method is based on geometrical assumptions that have certain limitations and could be unreliable when applied at the segmental level. The segmental data currently presented should therefore be considered as a velocity-displacement loop of the respective wall segment rather than being interpreted as estimates of regional flow-volume relationships. Secondly, it has to be emphasized that, in the current experiments, left ventricular volume was not calculated but, instead, time-dependent left ventricular area was measured and then employed to calculate its first derivative in order to obtain an estimate of time-dependent volume, i.e. flow. It has to be remembered, however, that area so measured may differ in its dynamic behavior (and then amplified in its first derivative) from corresponding volume data, although both still reflecting the same underlying (patho-) physiological processes. Consequently, it can hardly be expected that the area and true volume data would share the same characteristics in full details, and in order to make the distinction between these two different variables, the area-based measures in this study are called flow-volume estimates. Thirdly, the presented methodological approach assumes image quality high enough to provide a clear visualisation of left ventricular endothelial border in order to ensure its adequate delineation by automatic border detection software without contamination with parts of any area outside the true left ventricular cavity perimeter. Having the examined individuals hold their breath resulted usually in sufficiently high image quality but in a limited number of cases the proper data acquisition was not possible in all echocardiographic views due to anatomical variations in thoracic cavity. Finally, the reliability of the presented method is dependent on the optimal definition of the region of interest. Given the fixed geometry of the ROI, much attention should be paid to its adequate positioning to ensure that the left atrium would not be included in the ventricular volumes as the mitral annulus moves toward the apex during systole. Similarly, the region of interest should not be made too wide to avoid inclusion of right ventricular cavity or pericardial regions in the left ventricular volume assessment.

The use of left ventricular volumes in clinical practice is well documented [8], with end-diastolic and end-systolic volumes often derived in clinical echocardiography examinations routinely. Several quantitative techniques have been used to develop graphs of frame by frame left ventricular volume representation throughout the cardiac cycle, including ventriculography [9], two-dimensional echocardiography, three-dimensional echocardiography [10], radio-nuclide techniques [11], and conductance catheter technique [12], just to name a few. However, these approaches offer little information regarding the isovolumic events and the dynamic relationship between left ventricular flow and volume, or suffer from their inherent invasiveness. To our knowledge, there have been hitherto no previous attempts to combine the estimates of left ventricular volume and left ventricular flow in a non-invasive way. This study presents therefore a new development in this field by providing non-invasive possibility to obtain continuous quantitative information about these variables throughout the entire cardiac cycle. In addition to these quantifiable indices, other features of flow-volume estimates may also be appreciated visually, including contribution of atrial filling, prominence of isovolumic events and the overall form of the estimated flow-volume loop as a qualitative measure in the process of pattern recognition in health and disease.

The present methodological approach to demonstrate the relationship between left ventricular flow events and dynamic changes in ventricular volume may provide a novel opportunity to better understanding of cardiac (patho-)physiology. The estimated flow-volume loops may offer an attractive approach to the evaluation of varying degree of both systolic and diastolic dysfunction. Calculation of time-dependent estimate of the aortic valve area throughout the ejection period (according to the formula Q = v_mean _A; v_mean _: mean velocity, A : aortic valve area) by dividing consecutive derivative values of left ventricular area (representing flow, i.e. Q) by the corresponding Doppler-derived aortic velocities may become another possible application. The method also has the potential to be used as a quick communication interface, when comparing loops obtained at different occasions or in different patients. When comparing estimated flow-volume loops among the patients or during the course of disease in any given patient, not only the shape of the loop but also its actual position in the diagram may provide valuable information.

A deeper understanding of the significance of the observed regional variations in the estimated segmental flow-volume loops would require more extensive studies, potentially in combination with invasive conductance catheter measurements. A new possibility to identify early segmental myocardial dysfunction may then materialise. The currently observed regional variation in the estimated loop form in healthy individuals fits in with the complex fibre architecture of the left ventricule [13-15]. Especially during the isovolumic phases when the transient left ventricular reshaping takes place [16-21], the deformation of the subendocardial and subepicardial layers has been shown to be asynchronous [22] which fact would most probably result in heterogeneous regional expression of isovolumic events. In fact, heterogeneous regional distribution of the isovolumic myocardial displacement has been observed in one of our previous studies [23] and currently presented estimated flow-volume loops disclose similar regional heterogeneity in this respect as well.

The ability of our approach to demonstrate the relationship of peak flow events to dynamic volume change of the left ventricle may be particularly attractive in a diseased population.

In the patients with severe left-sided valvular abnormalities, marked changes were seen in the estimated flow-volume relationships in systole (aortic stenosis), and in diastole (mitral stenosis and aortic insufficiency). Although the changes noted could be intuitively anticipated, the morphology of the estimated flow-volume loops could reflect the nature and severity of the underlying valvular pathology. In the case of aortic stenosis, the observed estimated maximal systolic flow occurring at lower than normally estimated left ventricular volume would indicate obstruction of the ventricular emptying that might be valvular or subvalvular. Similarly, the estimated maximal diastolic flow at higher estimated diastolic volume in the case of mitral stenosis reflects most probably restricted ventricular filling and a greater contribution of active filling during atrial contraction, or widening of the mitral orifice during late diastole. Finally, the complex pattern of the diastolic section of the estimated flow-volume loop in the patient with aortic regurgitation results most probably partly from rapid filling of the left ventricle in early diastole, and partly from equivalent of the Austin Flint phenomenon [24] on the mitral valve opening.

## Conclusion

In conclusion, non-invasive estimation of left ventricular flow-volume characteristics throughout the cardiac cycle using transthoracic echocardiography is technically feasible and offers physiological information that has not hitherto been readily available. The presented estimates of flow-volume loops reveal the existence of regional morphological variation that, if explored further, may lead to a deeper understanding of the complex physiology of the left ventricle and it would be of particular interest to see whether the segmental alteration of flow-volume loop estimates could identify coronary stenosis that is by and large a segmental (regional) disease. Finally, severe left-sided valvular abnormalities cause a clear and specific alteration of the estimated normal systolic or diastolic flow-volume relationships that can be easy identified, supposedly reflecting the underlying hemodynamics specific for such abnormalities.

## Competing interests

The author(s) declare that they have no competing interests.

## Authors' contributions

LÅB initiated and designed the study. BL and PC performed all of the UL measurements. ES made all data conversions, plots and calculations from ultrasound data. BL, PC, JN and ES performed all the statistical analysis of the study. JN was responsible for the manuscript. All authors contributed to, read and approved the final manuscript.
